# Anti-cancer binary system activated by bacteriophage HK022 integrase

**DOI:** 10.18632/oncotarget.25512

**Published:** 2018-06-08

**Authors:** Amer Elias, Natasha Gritsenko, Rena Gorovits, Itay Spector, Gali Prag, Ezra Yagil, Mikhail Kolot

**Affiliations:** ^1^ Department of Biochemistry and Molecular Biology, Tel-Aviv University, Tel-Aviv 69978, Israel; ^2^ Institute of Plant Sciences and Genetics in Agriculture, Robert H. Smith Faculty of Agriculture, Food and Environment, The Hebrew University of Jerusalem, Rehovot 76100, Israel

**Keywords:** cancer therapy, site-specific recombination, coliphage HK022 integrase, lung cancer, DTA toxin

## Abstract

The binary system presented in this work is based on the bacteriophage HK022 integrase recombinase that activates the expression of a silenced Diphtheria toxin gene, both controlled by the cancer specific *hTERT* promoter. Using a lung cancer mice model, assays of different apoptotic and anti-apoptotic factors have demonstrated that the Integrase based binary system is highly specific towards cancer cells and more efficient compared to the conventional mono system whose toxin is directly expressed under *hTERT*. In a mice survival test, this binary system demonstrated longer persistence compared to the untreated and the mono treated ones. The reason underlying the advantage of this binary system over the mono system seems to be an overexpression of various *hTERT* suppressing factors induced by the mono system.

## INTRODUCTION

Gene therapy has become an important developing field in cancer treatment using DNA, RNA or oligonucleotides [1] and several technologies in this field are currently in advanced stages of clinical trials [2]. The main advantage of cancer gene therapy approaches is the use of targeted delivery classified by two different strategies: passive targeting and active targeting. Enhanced permeation and retention (EPR) is the basis of passive cancer targeting that is widely applied in numerous drug delivery systems for cancer targeting [3, 4], whereas in active targeting the basic concept is to utilize molecular targeting agents to specifically deliver the biomarkers or receptors towards the cancer cells thereby avoiding damage to normal tissue [5, 6]. However, the development of these tools still represents significant challenges [7]. Toxin therapy, as a part of cancer gene therapy, is based on toxic gene expression targeted exclusively to the cancer cells that result in killing through apoptosis without affecting healthy cells [8]. One of the most used toxins is the Diphtheria toxin subunit A (DTA) which carries the catalytic domain of the full-length Diphtheria toxin of *Corynebacterium diphtheria* [9–11]. DTA has a very efficient killing activity rate where a single DTA molecule is sufficient to kill the cell [12, 13]. Former studies have demonstrated selective expression of DTA in tumor cells directly downstream to cancer specific promoters [14, 15]. The main drawback of these mono systems is their incomplete specificity towards cancer cells due to residual cytotoxic expression also in healthy cells [16, 17] and the use of a site-specific recombinase (SSR) can overcome this. Harnessing of SSRs for genome manipulations and gene therapy has been a known approach for the past 20 years [18–22]. These systems encode recombinases that catalyze a site-specific recombination reaction between two specific short DNA sequences of 20-40 base pairs (bp) that serve as recombination sites (RSs) [23]. The SSR Integrase (Int) of coliphage HK022 (HK022) catalyzes phage integration and excision into and out of its *Escherichia coli* host chromosome whose site-specific recombination reaction mechanism is identical to that of the well documented Int SSR of coliphage Lambda [24, 25]. HK022's RSs are the bacterial attachment site *attB* and the phage attachment site *attP*. Integrative *attB* x *attP* recombination leads to phage integration into the host genome resulting in a prophage that is flanked by the recombinant *attL* and *attR* sites. The reverse *attL* x *attR* excision reaction is likewise catalyzed by Int. It was demonstrated that Int of HK022 catalyzes the integration and excision reactions in human cells [26, 27].

**Figure 1:**
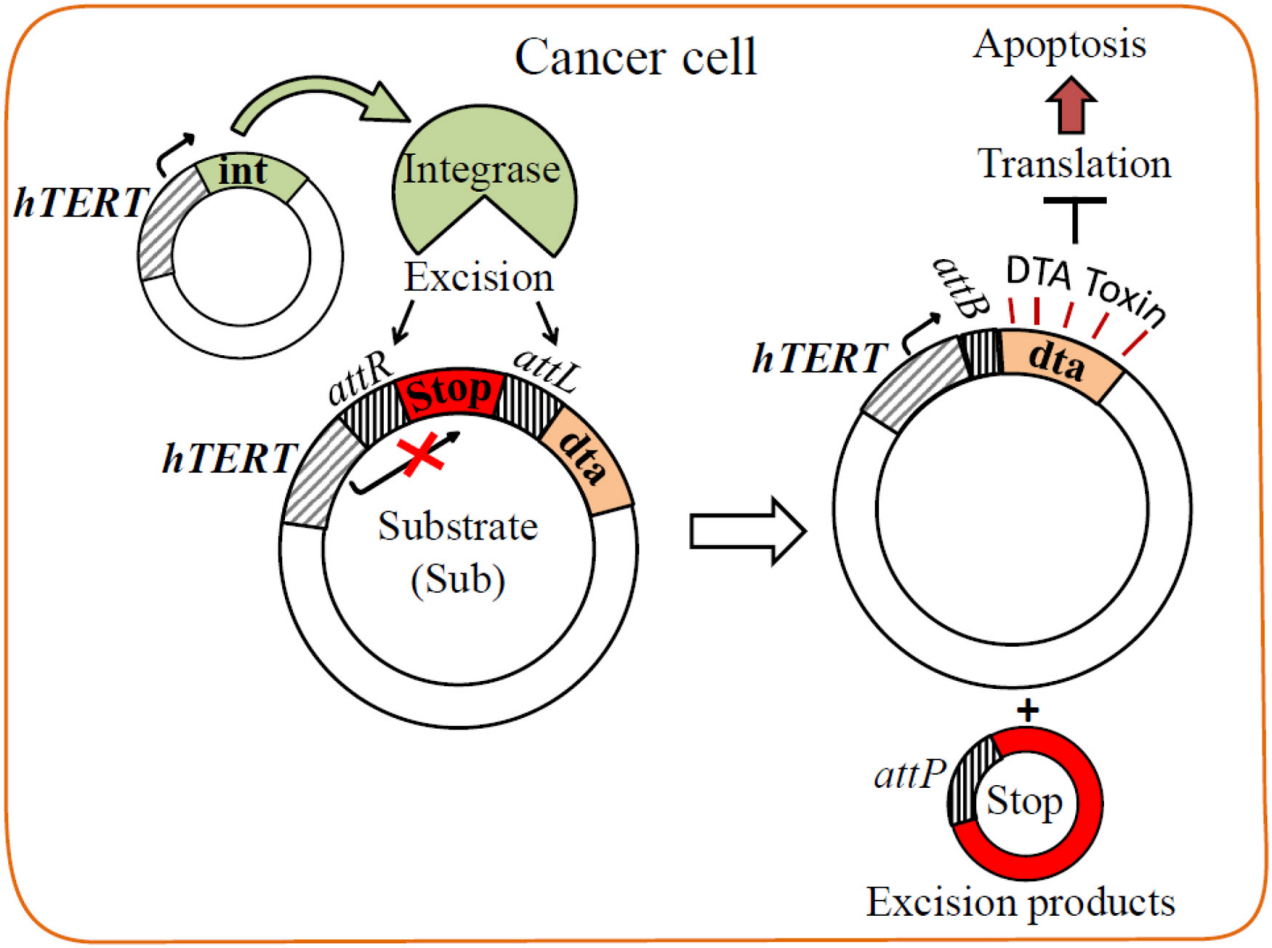
Scheme of the Int-based binary system The substrate plasmid carries a silenced DTA toxin gene separated from its *hTERT* promoter by a transcription terminator (Stop) flanked by *attR* and *attL* recombination sites (pAE1850). The excision reaction catalyzed by the *hTERT*-promoted Int (plasmid pNA1263) excises Stop and leaves the short *attB* site in the DTA expressing *hTERT*-*attB*-*dta* plasmid.

SSR`s are used in binary systems as an effective potential approaches for targeted cancer gene therapy (Figure 1, shown for Int). In these two-plasmid systems, one carries a silenced toxin gene separated from its promoter by a transcription terminator (Stop in Figure 1) and the second plasmid carries the recombinase under a cancer specific promoter. A site-specific recombination reaction activates the specific expression of the toxin in cancer cells. The SSR Cre was used in such binary system and proved enhanced specificity [16, 28]. Using luciferase as a reporter, we have recently developed an Int-based binary expression system and demonstrated its specificity in *human* cancer cells and in lung cancer mice [29]. Here we demonstrate the application of this HK022 Int-based binary system that, by a site-specific recombination reaction, induces DTA toxin expression that specifically kills tumor cells. This system has shown high safety and promising efficiency in mice. Possible advantages of this Int-based binary system over the other recombinases are discussed.

## RESULTS

### Integrase-based binary DTA expression system for specific cancer cell killing

Results of the Int-activated cancer-specific binary cell expression system using *luc* reporter [29] has motivated us to test its validity in killing specificity of cancer cells and in cancer mice using the DTA toxin. This DTA-based binary system (henceforth the binary system) consists of the following two plasmids (Figure 1). In the first, *int* is controlled by the cancer-specific *hTERT* promoter that is active in over 85% of tumors and immortal cells, but silent in somatic tissues [30, 31]. The second plasmid is Int's substrate plasmid (henceforth Sub) which carries the silenced open reading frame of the less active *dta* mutant (G128D) [32] that is separated from the *hTERT* promoter by a transcription terminator (Stop) that blocks DTA expression [33]. Stop is flanked by tandem *attR* and *attL* excision sites. Int-catalyzed *attR* x *attL* recombination excises the terminator sequence, thereby allowing expression of the toxin by the promoter. In this binary system, *hTERT* promoter controls the expression of both genes (*int* and *dta*).

### Efficiency and specificity of the binary system in tissue cultures

**Figure 2:**
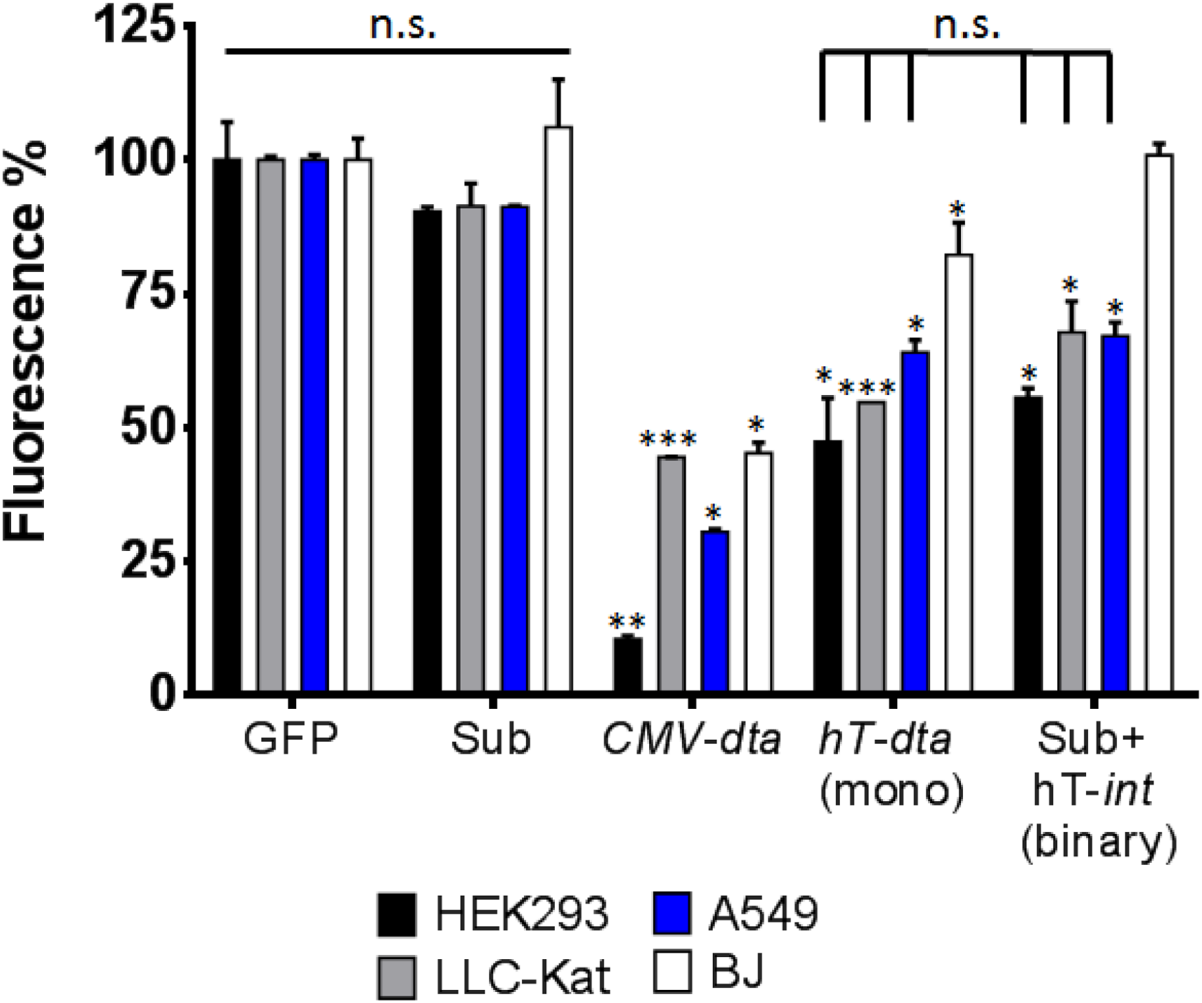
DTA toxicity assay *in vitro* HEK293, LLC-Kat, A549 and BJ cells lines were cotransfected with the GFP expressing plasmid (pEGFP-N1) along with the following plasmids: Sub (pAE1850); *CMV-dta* (pAE1808); *hTERT-dta* (mono, pNG1805); Sub+*hTERT-int* (binary), each plotted as percent of cells transfected with the GFP plasmid alone. GFP fluorescence was monitored by FACS. The bars show the mean values of four experiments; the error bars indicate standard deviation. n.s: not significant; ^***^p-val ≤ 0.001; ^**^p-val ≤ 0.01; ^*^p-val ≤ 0.05 vs. GFP alone.

The efficiency and specificity of the binary system DTA toxicity in various cell cultures were assayed using GFP expression as indicator [34]. Each cell line was cotransfected with the GFP reporter plasmid pEGFP-N1 along with the binary system. In parallel, cells of each line were cotransfected with the GFP plasmid along with each of the following three controls: the silent *dta* Sub by itself, a plasmid that expresses DTA under the strong *CMV* promoter and a plasmid that expresses DTA under the *hTERT* promoter (the mono system). In a fourth control, cells of each line were transfected with the GFP reporter plasmid alone to assess the transfection efficiency (as 100% fluorescence) as described in Materials and Methods. Toxicity of DTA was monitored by comparing GFP expression between the various treatments in surviving cells 48 hours post transfection using FACS analyses. The cell lines that each underwent these five transfections were HEK293, an immortalized human embryonic kidney cell line that expresses a high level of hTERT sufficient to maintain their telomeres indefinitely [35, 36], LLC-Kat cell line composed of Lewis lung carcinoma cells labelled with a red fluorescence gene [37] (these cells were used in our previous and current work to develop lung-metastatic tumors in mice [29]), A549 cell line consisting of human lung carcinoma cells and the BJ healthy cell line composed of normal human foreskin fibroblast cells. The first three lines (HEK293, LLC-Kat and A549) are all expected to respond positively to the binary system and the fourth line (BJ), consisting of normal cells, was used to examine the cancer specificity of the *hTERT* promoter. Results plotted as percent fluorescence of the cells treated with the GFP plasmid alone are presented in Figure 2. With the silent Sub alone all four lines showed no toxicity and were not significantly different from the positive 100% GFP control (91-106%, p-value≥0.05). All cell lines transfected with *CMV*-*dta* plasmid show a significant toxicity. Likewise, though less so, with the transfected *hTERT*-*dta* mono system plasmid including the non-cancerous BJ cell line. In contrast, with the binary system (Sub+*hT-int*) only the three expected cell lines showed a significant toxicity whereas the healthy BJ cells did not show any toxicity (100%). Though the DTA toxicity values of both mono and binary systems were similar in the cancer cell lines, in the normal BJ cells the binary system, unlike the mono system did not show any toxicity.

### The efficiency and specificity of the binary system in lung cancer mice

**Figure 3:**
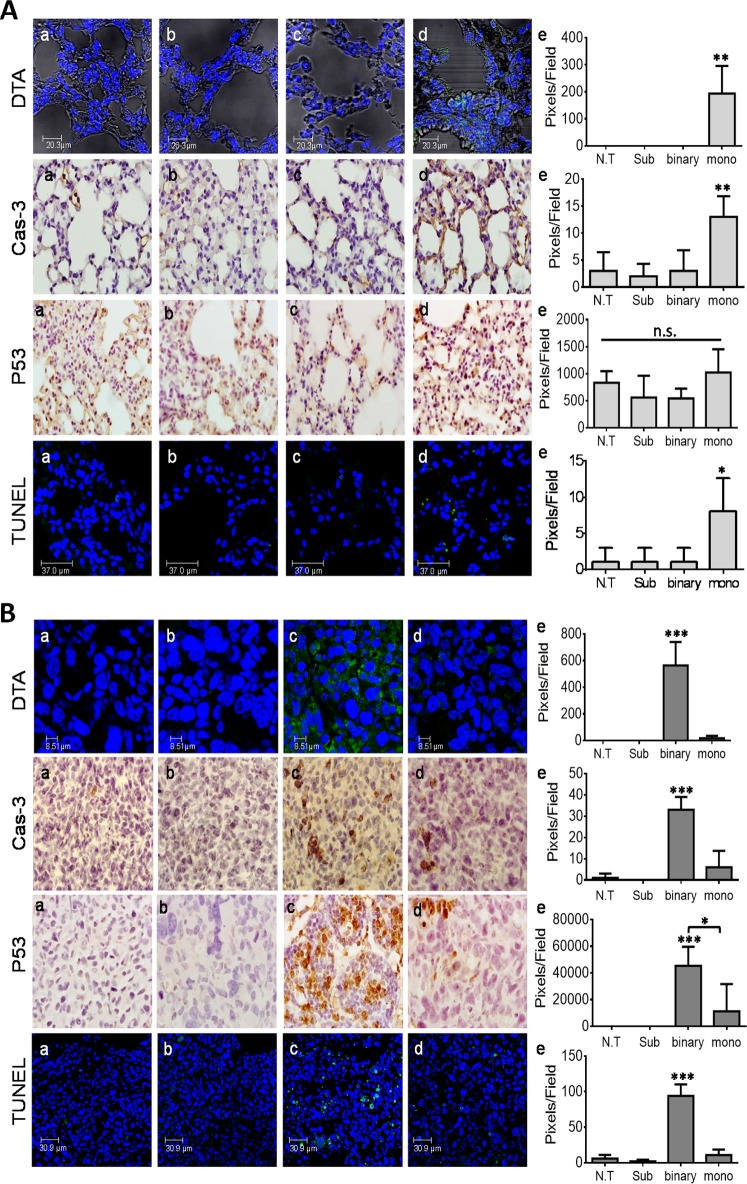
IHC analyses of mice lungs **(A)** Healthy lungs. **(B)** LLC-Kat lungs. DTA toxin (green spots), Cas-3, P53 (both brownish pigment) and TUNEL assay (green spots). (a) untreated mice (N.T.); (b-d), IV-tail injected mice with the following plasmids complexed with the jetPEI: (b) Sub (40μg), (c) binary (40μg Sub + 10μg *hTERT*-*int*), (d) mono (40μg *hTERT*-*dta*), (e) quantitative data. Each figure shows a typical lung biopsy specimen from a cohort of at least five mice. The bars show the mean value of five experiments; the error bars indicate standard deviation. n.s: not significant, ^***^p-val ≤ 0.001; ^**^p-val ≤ 0.01; ^*^p-val ≤ 0.05 vs. N.T; ^*^p-val ≤ 0.05 vs. *hTERT*-*dta*.

To examine the binary system cancer killing specific efficacy in whole organism cells *in vivo*, healthy and LLC-Kat cancerous mice where each divided into 4 groups (6 mice/group). One group remained untreated (a), in the other three groups, the mice were IV-tail injected with the following plasmid(s) complexed with the jetPEI delivery reagent: Sub plasmid alone (b), the binary system plasmids (c) and the mono system plasmid (d). Biopsy specimens of lung tissues were collected 24 hours post injection and underwent immunohistochemistry (IHC) analyses (Figure 3). In the first analysis, the presence of DTA was assessed by using DTA antibodies (Figure 3A, 3B, DTA, panels a-c). Though DTA was not expected to show in any of the treated healthy mice, the ones treated with the mono system showed low, but significant expression of the DTA (Figure 3A, DTA, panels d, e). In contrast, the ones treated with the binary system did not show any DTA (Figure 3A, DTA, panels c, e). Furthermore, in cancer lung cells the binary system showed a stronger DTA expression (Figure 3B, DTA, panels c, e), 33 folds higher than did the mono system (panel d). These results demonstrate the stronger efficiency of the binary system over the mono system and a complete specificity as compared to the healthy cells (Figure 3 panels d, e). Since DTA expression induces apoptosis [12, 13] the presence of three apoptotic markers was analyzed by IHC: Cas-3 that is most extensively studied as an early stage apoptotic protein among caspase family members [38]. P53 that is a key regulator of the cell cycle and is also known to function as an apoptotic marker [39] and TUNEL that is a common assay to identify final stages of apoptotic DNA fragmentation [40]. In accordance with the DTA data, the Cas-3 and TUNEL IHC tests of the healthy lungs treated with the mono system showed some but significant apoptosis (Figure 3A, Cas-3 and TUNEL panels d, e). On the other hand, the binary system showed a basal presence (panels c, e). Since these basal levels in panels b and c are also apparent in the untreated cells (panels a) they can be considered as an intrinsic background expression. In the case of P53, the healthy cells showed basal levels in all treatments as in the untreated group, probably due to its more common regulatory properties (Figure 3A, P53, panels a-e). However, in the cancer lungs (Figure 3B, P53), as with the other apoptotic markers, there was some apoptosis with the mono system but a significant apoptotic increase with the binary system and none with the Sub alone. In total, the data in Figure 3 confirm the remarkable stronger activity and a tight specificity of the binary system towards cancer cells.

**Figure 4:**
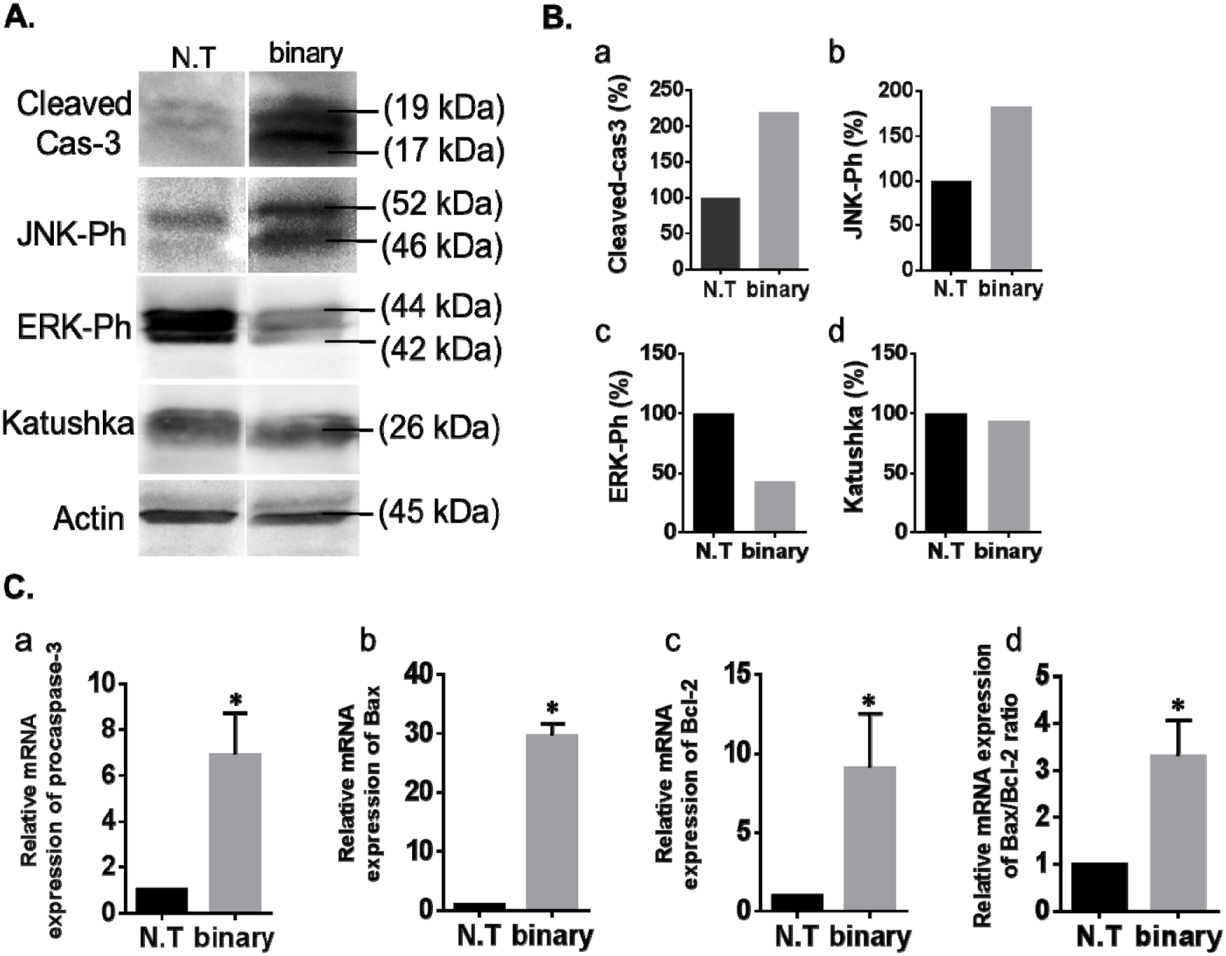
Western blots and qRT PCR analysis of the binary system in cancer lungs **(A)** Western blot analyses of apoptosis-associated and proliferation proteins in lungs of the LLC-Kat mice treated with the binary system as in Figure 3. Total lung cellular proteins extracted from LLC-Kat lungs were analyzed using antibodies recognizing the following apoptotic and proliferation markers: cleaved Cas-3, Ph-JNK, Ph-ERK, Katushka and Actin. The latter marker was used as unrelated controls. Each panel shows the typical lungs from a cohort of at least three mice. **(B)** Western blots quantitative data. N.T. – untreated lungs. binary – lungs treated with Sub+*hTERT*-*int*. **(C)** qRT PCR analyses of RNA extracted from lungs of LLC-Kat mice treated with the binary system analyzing the expression of the following genes: (a) procaspase-3, (b) Bax, (c) Bcl-2. Expression levels were normalized to Katushka mRNA level. (d) Bax to Bcl-2 ratio. ^*^p-val ≤ 0.05 vs. N.T.

To further support the binary system efficacy in cancer lungs additional apoptotic, anti-apoptotic and tumor specific markers were examined by western blots and qRT PCR analyses. Cellular proteins were extracted from the lungs and western blots were performed using antibodies against the following apoptotic protein markers: Cas-3, activated/phosphorylated Jun N-terminal kinase (JNK-Ph) [41, 42], the anti-apoptotic phosphorylated mitogen-activated protein kinase (ERK-Ph) [43, 44] and as positive controls the Katushka fluorescent reporter and Actin. Figure 4A, 4B show, as expected, that the levels of both apoptotic markers Cas-3 and JNK-Ph increased abundantly in the lungs of the treated mice and that of the ERK-Ph proliferation marker was decreased. The levels of the Katushka patterns were evident in both cancer lung samples, confirming their cancer development. In addition, Figure 4C, panels a-c presents a qRT PCR analysis of the following apoptotic genes showing the effect of the binary system in mini-transcriptomes: *Cas-3*, *bax* that is functionally characterized as an apoptotic-promoting factor and *bcl-2* that is characterized as an apoptotic suppressing factor [45, 46]. In all three cases, the lungs treated with the binary system showed a significant elevated expression of these genes. Despite the fact that Bcl-2 is known as an anti-apoptotic factor, it has been well documented that while apoptosis the expression of Bcl-2 does increase [47]. To testify apoptosis, the *bax/bcl-2* expression ratio must be increased as being the cellular 'rheostat' of apoptotic sensitivity [48, 49] and this is the case with the binary treated lungs (Figure 4C, 4d).

### Effect of the binary system in a survival test

**Figure 5:**
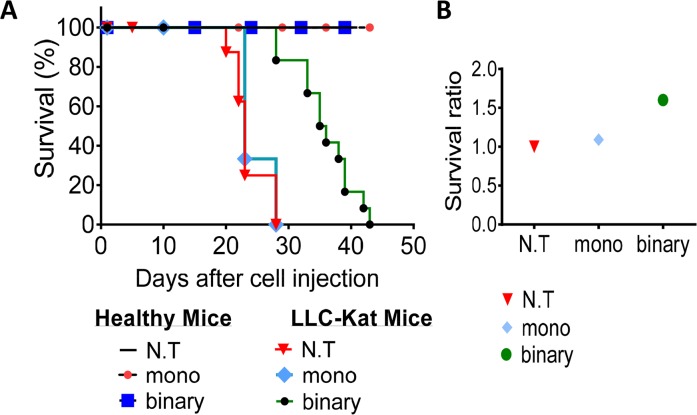
Survival experiment **(A)** Kaplan-Meier survival plot of healthy and LLC-Kat mice treated with the binary system (Sub+*hTERT*-*int*), the mono system (*hTERT*-*dta*) and of untreated mice (N.T.). **(B)** Survival ratio of the LLC-Kat cancer mice treated with the binary system, mono system and untreated mice. The mice were retro-orbital injected (after positive detection of lung cancer metastases by CT analysis) systemically each 3-4 days with the binary system (Sub+Int, 50μg+10μg respectively) or the mono system (50μg *hTERT*-*dta*) plasmid DNA complexed with jetPEI. Each group presents a cohort of n≥6 (n – mice number).

In the following experiment (Figure 5), we compared the survival efficacy of lung cancer mice and healthy mice treated with the binary system, the mono system along with untreated controls (≥6 mice/group). Retro-orbital plasmid-jetPEI complex injections (day 0) commenced 10 days after the LLC-Kat cells injections of the cancerous mice whose lung cancer metastases were pre-confirmed by CT analyses. These complexed DNA injections were systemically repeated thereafter each 3-4 days. As shown in Figure 5A all healthy mice survived the DNA injections whereas among the cancerous mice the untreated and mono system treated groups death commenced at days 22 and 25, respectively, and by day 28 none survived. In contrast, among the binary system treated group death commenced a week later (day 29), it continued less abruptly and the last individual of this group died on day 43, significantly later and slower than the other two groups. Figure 5B shows a survival ratio of 1.6 folds of the binary treated over the untreated control cancer mice; while there was no real difference between the mono-treated mice and the untreated ones.

To test again the apoptotic markers at the end of this survival experiment biopsy specimens of lungs tissues were collected (post mortem in case of cancer mice or day 45 in the case of healthy mice) and used for IHC and qRT PCR analyses (Supplementary Figure 1). In healthy mice lungs, the binary system effect has confirmed its high safety by showing only basal levels of Cas-3 and P53 as in the untreated group together with a complete absence of TUNEL activity (Supplementary Figure 1A, panels a, b, d). However, the mono system showed a significant induction of Cas-3 and TUNEL activity compared to untreated mice (Supplementary Figure 1A, panels a, c, d) that was much stronger than the data in Figure 3 due to systemic treatments. In the cancer lungs, the binary system has confirmed its high apoptotic efficacy by the elevated Cas-3, P53 and TUNEL activity (Supplementary Figure 1B, panels a, b, d) that for Cas-3 and TUNEL were also much stronger compared to Figure 3. In addition, an examination of the tumor-associated macrophage marker F4/80 was also conducted [50, 51] that like the other markers showed a significant increase in expression. Supplementary Figure 1C (a-c) show the results of the lung mini-transcriptomes of the *Cas-3*, *Bax* and *Bcl-2* genes analyzed by qRT PCR. As before, in the cancer lungs, biopsy specimens treated with the binary system all three genes showed a significant three to nine folds expressed elevation compared to the untreated group. The *bax/bcl-2* ratio showed a two folds increase (Supplementary Figure 1C, 1d). Overall, the data from cancer mice of the survival experiments treated with the binary system show substantial increased apoptotic activities, a four to seven folds elevation compared to the mono system (Supplementary Figure 1B panels b-d) and that for some of the apoptotic marker are stronger than in the 24-hours treated mice (Figure 3), reconfirming the stronger activity and safety of the binary system over the mono system.

It was also important to examine the safety of the binary and mono systems in the spleen of healthy mice due to the facts that *hTERT* promoter is active also in the spleen [52, 53] and that the retro-orbital jetPEI-DNA complex injection is also delivered to the spleen (Polyplus database). Supplementary Figure 2, panels, a, b, d show that the biopsy specimens of the binary system-treated mice show no or basal level of Cas-3, P53 and TUNEL assay. However, in the ones treated with the mono system the Cas-3 and TUNEL showed a substantial induction (Supplementary Figure 2, panels c, d), as with the lungs (Supplementary Figure 1A). These data confirm that despite the potential leakage of the binary system into the spleen it retains its safety.

### Exploring the molecular mechanisms underlying the efficacy killing superiority of the binary system over the mono system

The survival experiment (Figure 5) has demonstrated that the binary system enabled an extended survival of the cancerous mice whereas the mono system did not. This may be explained by differences in the regulation by the *hTERT* promoter due to the supply of plasmids that carry this promoter. In more than 85% of tumor cell types hTERT expression is upregulated leading to constitutive telomerase activity that is critical for tumorigenesis [54]. This indicates that telomerase activity is required for the long-term viability of tumor cells. The *hTERT* and the mouse *TERT* (*mTERT*) promoter sequences are highly preserved and both contain various binding sites for different transcription factors such as members of the Myc/Max/Mad family and E2F1 that take part in cellular processes including proliferation, differentiation and apoptosis [55, 56]. Among them Mad1, E2F1 and Smad3 (a member of the Smad family) were identified as transcriptional suppressors in which the first two are dependent on the c-Myc pathway and the third is a c-Myc independent pathway [57]. It is plausible that under our experimental conditions, the presence of a high molecular number of active exogenous *hTERT* promoters located on the delivered plasmids may distort the normal regulation balance of the mouse native *mTERT* promoter. With the mono system, the excess of exogenous *hTERT* promoters (50 μg plasmid/mouse) presumably leads to an immediate need to preserve the normal balance of mTERT by over expression of various transcription inhibition factors like Mad1, E2F1 and/or Smad3 in order to suppress these imported exogenous *hTERT* promoters. In the case of the binary system, the *hTERT* promoter on the Sub plasmid (50 μg plasmid/mouse) is inert due to the intervention of the inserted transcription terminator (Stop) that arrests polymerase activity and avoids the need to bind transcription inhibition factors. Thus, and in contrast to the mono system, the normal balance of TERT in these cells is not affected and an elevation of the transcription inhibition factors is less expected, both in healthy as well as in cancer cells. In addition, because Int is less active than are other SSRs like Flp and Cre [58] and (unpublished data), its weaker recombination activity leads to the formation of only a small amount of the active *hTERT*-*attB*-*dta* recombination product in LLC-Kat cancer cells (Figure 1) which does not affect the normal TERT balance such that no excess of *TERT* suppressors is needed. Nevertheless, this small amount of the active recombination product that expresses the highly efficient DTA toxin suffices to kill the cancer cells.

**Figure 6:**
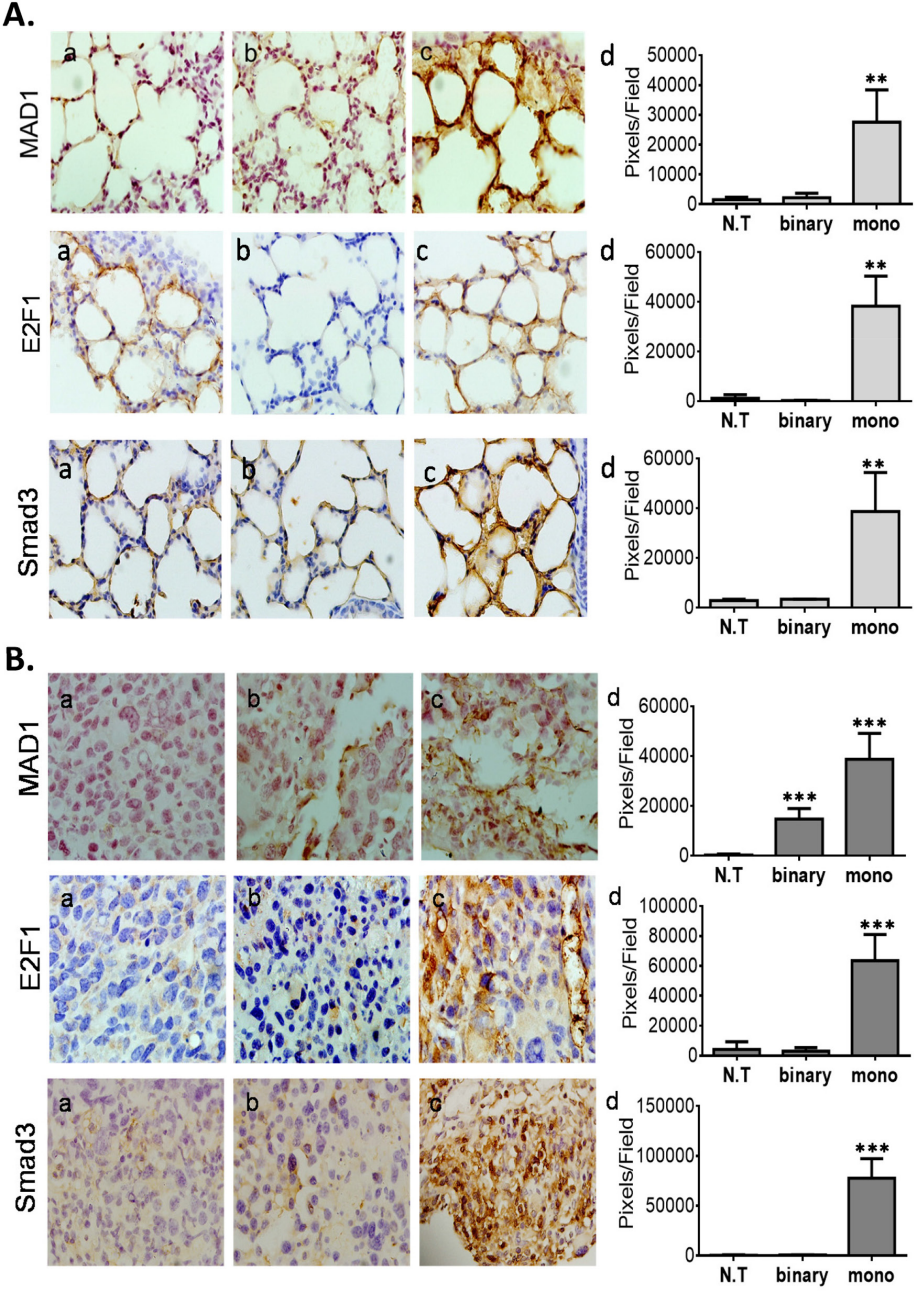
IHC analyses of *TERT* promoter regulatory suppressing factors of the survival experiment mice **(A)** Healthy lungs. **(B)** LLC-Kat lungs. Mad1, E2F1 and Smad3 (all brownish pigment). (a) untreated mice (N.T.); (b) binary, (c) mono, (d) quantitative data. Each figure shows a typical lungs biopsy specimen from a cohort of at least six mice. The bars show the mean value of five experiments; the error bars indicate standard deviation. ^***^p-val ≤ 0.001; ^**^p-val ≤ 0.01vs. N.T.

To test this suggestion we analyzed by IHC the expression levels of Mad1, E2F1 and Smad3 in healthy and cancerous mice lungs of the survival assay (Figure 6A, 6B, respectively). Lung IHC analyses of both mice types has revealed that only the mono system showed substantial increased levels of Mad1, E2F1 and Smad3 (Figure 6, panels c, d). The conspicuous induction of these suppressors only in the mono system-treated healthy and cancerous mice support our hypothesis that this is aimed to down regulate excess active intracellular *hTERT* sequences. Notably, some Mad1 elevation was also evident in the binary system (Figure 6B, panels b, d), though three folds lower than in the mono system (Figure 6B, panels c, d). The reason for this lower but significant elevation of Mad1 might be due to the presence of the systemically supplied *hTERT*-*int* promoted plasmid that is part of the binary system, carries active *hTERT* sites and has also catalyzed some of the Substrate plasmid to form additional active *hTERT* products. Finally, the expression of the three suppressors in the cancer mice (Figure 6B) is 1.4-2.0 folds higher than in healthy ones (Figure 6A). This may explain why in the mono system-treated mice shown in Supplementary Figure 1 the expression of Cas-3 was higher in the less suppressed healthy mice (Supplementary Figure 1A) than in the cancerous ones (Supplementary Figure 1B). Moreover, in 24-hours IHC assay (Supplementary Figure 3) the expression of Mad1 and E2F1 was, respectively, six and two folds higher in the mono system-treated cancer mice (Supplementary Figure 3B) compared to the healthy ones (Supplementary Figure 3A). This may explain why in the mono system-treated mice shown in Figure 3 the expression of DTA and Cas-3 were higher in the less suppressed healthy mice (Figure 3A) than in the cancerous ones (Figure 3B).

## DISCUSSION

In this work, we have developed an anti-cancer binary system based on a site-specific recombination reaction catalyzed by the HK022 Int recombinase that activates the expression of the *dta* toxin gene specifically in lung cancerous cells. Overall, unlike the mono system the binary system has demonstrated its safety towards normal cells (Figures 3A and Supplementary Figure 1A) and its substantially higher efficiency in cancer cells (Figures 3B and Supplementary Figure 1B). The reason for the high specificity of the binary system towards cancer cells seems to be the combination of a safe inactive substrate and the low Int activity that suffices to catalyze the formation of a low amount but strongly active DTA. These might render Int advantageous over Cre and Flp in these systems. Moreover, Cre-dependent cellular toxicity has been demonstrated in eukaryotic cells and whole organism [34, 59] whereas the data in this work and the previous one [29] did not show any apoptotic effect by Int in healthy mice (Figures 3A, Supplementary Figures 1A and Supplementary Figure 2). In contrast to the mono system, the results have shown that the binary system does not affect the intracellular *hTERT* regulation (Figure 6), which may be another reason for its stronger activity.

Although the Int-based binary system compared to the mono system has demonstrated a stronger anti-cancer efficiency, yet in the survival experiment, the cancer mice treated with the binary system did not survive longer than 43 days (Figure 5). That is probably because the starting point of the treatment commenced at a relatively advanced stage of the disease (10 days after the LLC-Kat injections) such that the killing activity of the binary system was not sufficient to cure the advanced metastases. Moreover, the systemic delivery of the binary system by retro-orbital injections caused irreversible changes in the injection region that might have reduced the delivery efficiency, especially in the later steps. Better diagnostic tests that discover earlier stages of the lung cancer such as luc bioluminescence imaging [60], small-molecular fluorescent probes [61] or contrast-enhanced computerized tomography [62] for earlier DNA deliveries may lead to an improved or complete survival. Moreover, a combination of the binary system with different anti-cancer approaches may enhance the longevity of the treated cancer mice. In this experiment the healthy group treated with the mono system did survive despite showing substantial elevations of apoptotic markers (Supplementary Figure 1A), testifying the incomplete safety of the mono system.

Another feature of the binary system technology used here is the non-viral jetPEI delivery reagent, which worked efficiently. Despite obvious advantages of the viral systems, they possess a number of drawbacks like insertional mutagenesis, cell toxicity, immunogenicity, and a pre-existing immunity. Non-viral gene therapy has become a favorable gene therapy tool owing to its potential to address many of these limitations, particularly with respect to safety [1, 2]. Other superiorities of the binary-jetPEI system are the absence of toxicity [29], lack of immune system response [63] and the absence of cancer cells acquired resistance against the DTA molecule. Moreover, DNA complexed with jetPEI does not induce any pro-inflammatory response because of systemic injections, especially when compared to highly immunogenic compounds such as lipid transfection reagents or branched PEI [64]. Projects based on jetPEI are currently in advanced stages of clinical trials [2].

Apart of the *hTERT* promoter, alternative cancer specific promoters identified for several cancer types can provide a satisfactory selective expression of the gene of interest in specific cancer cells [65, 66]. To our knowledge, no comparative analysis has been reported to identify the functions of different promoters in context of binary systems for different types of cancer. Moreover, the possibility of combinatorial approaches of the binary system using two different cancer specific promoters of the targeted cancer may substantially expand the capabilities of this technology by better specific killing.

## MATERIALS AND METHODS

### Cells, growth conditions, plasmids and oligomers

The bacterial strain used was *E. coli* K12 TAP114 (*lacZ*) ΔM15 [67]. It was grown and plated on Luria-Bertani (LB) rich medium with the appropriate antibiotics. Plasmid transformations were performed by electroporation [68]. Plasmids and oligomers are listed in Tables 1 and 2, respectively.

**Table 1:** List of the plasmids

Plasmid	Relevant genotype*	Source
pUC18	ApR	Clontech
pEGFP-N1	*CMV-GFP*, KmR	Clontech
pMK189	*CMV*-*attR*-Stop-*attL*-*GFP*, KmR	[70]
pKS1161	*CMV*-*attR*-Stop-*attL*-*dta*, KmR	This work
pNA1263	*hTERT*-*int* expressing plasmid, ApR	[29]
pNG1805	*hTERT*-*dta*, ApR	This work
pAE1808	*CMV-dta*, KmR	This work
pAE1850	*hTERT*-*attR*-Stop-*attL*-*dta*, ApR	This work

**Table 2:** List of the primers

Primer number	Gene	Primer sequence
557-f	*dta*	TACCGGTTCCACTAGTCCAGTGTGGTGGAAT
558-r	*dta*	AGCGGCCGCAGGTTCCTTCACAAAGATCGCCTGAC
1060-f	Katushka	CTCAAGATGCCCGGCGTCTAC
1061-r	Katushka	CCTCGTGCTGCTCGACGTAG
1149-f	Katushka	CCCTGAGGGCTTCACATGGGAGAGG
1148-r	Katushka	CCCCAGTTTGCTAGGCAGGTCGC
1124-f	Bax	CCGGCGAATTGGAGATGAACT
1125-r	Bax	CCAGCCCATGATGGTTCTGAT
1152-f	Bax	GCGGGCCCACCAGCTCTG
1153-r	Bax	GACAAGCAGCCGCTCACGG
1137-f	Cas-3	CTGGACTGTGGCATTGAGAC
1138-r	Cas-3	GCAAAGGGACTGGATGAACC
1151-f	Cas-3	CCAACCTCAGAGAGACATTCATGGGCCTG
1150-r	Cas-3	GTGGAGTCCAGGGAGAAGGACTCG
1158-f	Bcl-2	GCTACCGTCGTGACTTCGC
1159-r	Bcl-2	TCCCAGCCTCCGTTATCC

Human embryonic kidney HEK293 and lung carcinoma A549 cells were cultured in Dulbecco's modified Eagle's medium (DMEM). Human normal skin fibroblast BJ cells were cultured in DMEM:M199 4:1 medium. Mouse Lewis lung carcinoma LLC1 cells labeled with the Katushka fluorescent gene reporter [69] (LLC-Kat) were cultured in RPMI medium. For transient transfection of HEK293, LLC-Kat, A549 and BJ, the cells (~6×10^5^) were plated in a 6 well plate and 24 h later treated with 2.3 μg of the proper plasmid DNA using CalFectin transfection reagent (SignaGen Laboratories, MD, USA) for HEK293 and TransIT-XT2 reagent (Mirus, WI, USA) for LLC-Kat, A549 and BJ. pEGFP-N1 transfected cells were used to estimate the transfection efficiency. The extent of GFP positive expressing cells detected by FACS was in HEK293: 75%, LLC-Kat: 26%, A549: 18%, BJ: 20%. All the experiments were repeated at least three times.

### Plasmid construction

Plasmid pKS1161 carrying *CMV*-*attR*-Stop-*attL*-*dta* cassette (Stop- transcription terminator [33]) was constructed by ligation of the AgeI-NotI *dta* PCR fragment generated from plasmid pIBI30-176 (kindly provided by Dr. F. Maxwell) as template and primers oEY557+oEY558 into the same sites of plasmid pMK189. Plasmid pNG1805 carrying the *dta* gene under the control of *hTERT* promoter was constructed by ligation of the AgeI-blunted-NotI *dta* fragment from pKS1161 with HindIII-blunted- NotI pNA1263 plasmid. The plasmid pAE1808 carrying the *dta* gene under the control of *CMV* promoter was constructed by ligation of the AgeI-NotI *dta* fragment from pKS1161 into the same sites of plasmid pEGFP-N1. Plasmid pAE1850 was constructed by ligation of the NheI-blunted-AgeI *attR*-Stop-*attL* fragment from pMK189 and the AgeI-NotI *dta* fragment from pAE1808 into HindIII-blunted-NotI pNA1263 plasmid. All plasmid constructs (Table 1) were verified by DNA sequencing.

### DTA toxin activity assay in cell cultures

To estimate Int-dependent DTA toxin activity, each cell line was cotransfected with the 1 μg GFP reporter plasmid (pEFGPN-1), 300 ng of the site-specific recombination substrate plasmid pAE1850 and 1 μg of the Int expressing plasmid pNA1263. As a positive control of 100% GFP expression, the cells were cotransfected with 1 μg pEFGPN-1 and 1.3 μg pUC18 as mock. As a positive control of DTA activity the cells were cotransfected with 1 μg pEFGPN-1, 300 ng of a plasmids carrying *dta*, either under the control of *CMV* (pAE1808) or under *hTERT* promoter (pNG1805) and 1 μg pUC18. As a negative control, cells were cotransfected with 1 μg pEFGPN-1, 300 ng of the substrate plasmid pAE1850 and 1 μg pUC18. All cotransfections were performed in total DNA quantity of 2.3 μg. The efficiency of toxin activity was estimated by FACS analysis of the GFP expressing cells that survived 48 hours post transfection.

### Fluorescent-activated cell sorting (FACS) analysis

~2 × 10^6^ cells from one well of a 6-well plate were collected following trypsin treatment of which 10^4^ cells were selected by the FACS sorter (Becton Dickinson Instrument) for fluorescent measurements of surviving cells. Data analysis was performed using the Flowing Software. Forward and side-scatter profiles were obtained from the same samples.

### DNA delivery in mice

DNA delivery in mice (strain C57BL/6) was performed using the linear polyethylenimine based delivery reagent *in vivo*-jet PEI (Polyplus transfection, France). In the 24 hours experiments, IV-injections were performed as previously described [29]. In the survival experiments, the DNA delivery was performed by retro-orbital injection. All mice procedures were performed in compliance with Tel Aviv University guidelines and protocols approved by the Institutional Animal Care and Use Committee.

### Lung cancer development and fluorescence imaging in mice

8 × 10^5^ LLC-Kat cells were IV tail-injected into 8 weeks' old (18–21 gr) female mice. Lung tumor metastases development was examined nine and twelve days following the injection using *in vivo* micro-CT scanner (TomoScope® *In-vivo* CT, Germany). The Katushka fluorescent signal in the lung metastases was analyzed on day 12 by an *ex vivo* fluorescence imaging system (CRi MaestroTM, USA) [29].

### Survival experiments

Healthy and LLC-Kat treated groups of mice (following positive detection of lung cancer metastases by CT analysis) were each divided into three treatment groups (cohorts of at least 6 mice/group). One remained untreated, the second (binary) injected with 50μg of the substrate plasmid pAE1850 + 10μg of the Int expressing plasmid pNA1263 all complexed with *in vivo*-jet PEI (jetPEI) and the third (mono) treated with 50μg of *hTERT*-*dta* plasmid pNG1805. The treatments were repeated each 3-4 days. At the end of the experiment (day 45), biopsy specimens from all three groups of healthy mice were collected from lungs and spleens, lungs of the LLC-Kat mice were collected post mortem.

### Preparation of sections, immunohistochemistry, confocal microscopy, apoptosis TUNEL assay and image analysis

Preparation of sections, immunohistochemistry, confocal microscopy, apoptosis TUNEL (terminal deoxynucleotidyltransferase-mediated dUTP nick end labeling) assay and image analysis were performed as previously described [29]. Lungs and spleens were immune-stained with anti-DTA (Cat#4701, ViroStat, Portland, ME, USA), cleaved Cas-3 (Cat# 9664S, Cell Signaling, MA, USA), P-53 (Cat# SC-6243, Santa Cruz Biotechnology, USA), Mad1 (Cat# 4682, Cell Signaling, MA, USA), E2F1 (Cat# MA5-14344, Thermo Fisher Scientific, Waltham, MA, USA), F4/80(Cat# ab6640, Abcam, Cambridge, MA, USA), Smad3 (Cat# 51-1500, Thermo Fisher Scientific, Waltham, MA, USA), Katushka (Cat# AB233, Evrogen, Moscow, Russia) antibodies at 1:200 dilution and with a TUNEL kit (MBL international, Woburn, MA, USA) according to manufacturer's instructions. Donkey anti-Goat IgG (H+L) secondary antibodies conjugated with Cy2 dye (Cat# 705-225-147, Jackson ImmunoResearch, PA, USA) was used for DTA detection. HiDef Detection HRP Polymer System (Cat# 954D-10, Cell Marque, CA, USA) was used for cleaved Cas-3, P53, Mad1, Smad3, E2F1 and Katushka detection. Pollnk-2 Plus HRP Detection Kit for Rat Primary Antibody (Cat# D46-6, GBI Labs, WA, USA) was used for F4/80 detection.

### Immuno-detection of cellular protein extracts

Mouse lungs were dissected and washed in CMF buffer (137 mM NaCl, 2.7 mM KCl, 8 mM Na_2_HPO_4_, 1.5 mM KH_2_PO_4_, 5.5 mM glucose). Then homogenized in lysis buffer (1 M sorbitol, 10 mm HEPES, 5 mm EDTA, 0.25 M NaCl, 0.2% Triton X-100, 0.2% NP40) and complete protease inhibitor mixture (Roche Applied Science) using 10 strokes of pestle A and 10 strokes of pestle B in a Dounce homogenizer. After 30 minutes incubation on ice with vortex, the protein extracts were cleared by spin-down, and a standard PAGE loading buffer supplemented with 2% SDS was added. Samples were incubated at 65°C for 15 minutes and subjected to 12% SDS PAGE. Immuno-detection of cellular proteins was performed by western blotting according to standard procedures. Antibodies used for western blotting included anti-cleaved Cas-3, Katushka, anti-phospho-SAPK/JNK, anti-phospho-ERK (Cat#9251S and Cat#9101S, Cell Signaling Technology, USA) and anti-actin (Cat#ab180, Abcam, Cambridge, MA, USA). Incubation with peroxidase-coupled secondary antibodies (Cat#SAB3700010 Sigma–Aldrich, USA) was followed by the enhanced chemiluminescence detection procedure (Amersham, Bucks, UK).

### Quantitative real-time PCR

Total RNA was isolated from murine lungs with the EZ-RNA Isolation kit (Biological Industries, Beit Haemek, Israel). cDNA was synthesized using the Verso cDNA kit (Applied Biosystems, Foster City, CA). mRNA expression was assessed by StepOne quantitative Real-Time PCR system (Applied Biosystems, Foster City, CA). As a quantitation method, we used the Relative Standard Curve. qRT-PCR of Katushka, Cas-3, Bax and Bcl-2 mRNA were preformed using the primers presented in Table 2. To calculate the molecular number of the markers (Katushka, Cas-3, Bax and Bcl-2), the concentrations were calculated using the formula: μg DNA x pmol/660 pg x 10^6^ pg/1 μg x 1/N = pmol DNA. The pmol were converted to mole by dividing by 1 × 10^12^, and then multiplied by 6.022 × 10^23^. The molecules number of Cas-3, Bax and Bcl-2 was divided by the molecules number of Katushka in order to normalize their gene expression to one Katushka molecule.

### DNA manipulations

Plasmid DNA from *E. coli* was prepared using a DNA Spin Plasmid DNA purification Kit (Intron Biotechnology, Korea) or a NucleoBond^TM^ Xtra Maxi Plus EF kit (Macherey-Nagel, Germany). General genetic engineering procedures were performed as described by Sambrook and Russell [68].

### Statistical analyses

Data were presented as the mean ± SD. *p*-values of less than 0.05 were considered statistically significant by Student's two-tailed t-test assuming equal variance.

## SUPPLEMENTARY MATERIALS FIGURES



## References

[1] Hardee CL, Arevalo-Soliz LM, Hornstein BD, Zechiedrich L (2017). Advances in non-viral DNA vectors for gene therapy. Genes.

[2] Yin H, Kanasty RL, Eltoukhy AA, Vegas AJ, Dorkin JR, Anderson DG (2014). Non-viral vectors for gene-based therapy. Nat Rev Genet.

[3] Maeda H, Wu J, Sawa T, Matsumura Y, Hori K (2000). Tumor vascular permeability and the EPR effect in macromolecular therapeutics: a review. J Control Release.

[4] Maeda H (2001). The enhanced permeability and retention (EPR) effect in tumor vasculature: the key role of tumor-selective macromolecular drug targeting. Adv Enzyme Regul.

[5] Byrne JD, Betancourt T, Brannon-Peppas L (2008). Active targeting schemes for nanoparticle systems in cancer therapeutics. Adv Drug Deliv Rev.

[6] Lee HY, Mohammed KA, Nasreen N (2016). Nanoparticle-based targeted gene therapy for lung cancer. Am J Cancer Res.

[7] Lattime E, Gerson S (2014). Gene Therapy of Cancer: Translational Approaches from Preclinical Studies to Clinical Implementation.

[8] Oldfield EH, Ram Z, Culver KW, Blaese RM, DeVroom HL, Anderson WF (1993). Gene therapy for the treatment of brain tumors using intra-tumoral transduction with the thymidine kinase gene and intravenous ganciclovir. Hum Gene Ther.

[9] Showalter SL, Huang YH, Witkiewicz A, Costantino CL, Yeo CJ, Green JJ, Langer R, Anderson DG, Sawicki JA, Brody JR (2008). Nanoparticulate delivery of diphtheria toxin DNA effectively kills Mesothelin expressing pancreatic cancer cells. Cancer Biol Ther.

[10] Amit D, Tamir S, Hochberg A (2013). Development of targeted therapy for a broad spectrum of solid tumors mediated by a double promoter plasmid expressing diphtheria toxin under the control of IGF2-P4 and IGF2-P3 regulatory sequences. Int J Clin Exp Med.

[11] Cocco E, Deng Y, Shapiro EM, Bortolomai I, Lopez S, Lin K, Bellone S, Cui J, Menderes G, Black JD, Schwab CL, Bonazzoli E, Yang F (2017). Dual-targeting nanoparticles for in vivo delivery of suicide genes to chemotherapy-resistant ovarian cancer cells. Mol Cancer Ther.

[12] Perentesis JP, Waddick KG, Bendel AE, Shao Y, Warman BE, Chandan-Langlie M, Uckun FM (1997). Induction of apoptosis in multidrug-resistant and radiation-resistant acute myeloid leukemia cells by a recombinant fusion toxin directed against the human granulocyte macrophage colony-stimulating factor receptor. Clin Cancer Res.

[13] Thorburn J, Frankel AE, Thorburn A (2003). Apoptosis by leukemia cell-targeted diphtheria toxin occurs via receptor-independent activation of Fas-associated death domain protein. Clin Cancer Res.

[14] Abdul-Ghani R, Ohana P, Matouk I, Ayesh S, Ayesh B, Laster M, Bibi O, Giladi H, Molnar-Kimber K, Sughayer MA, de Groot N, Hochberg A (2000). Use of transcriptional regulatory sequences of telomerase (hTER and hTERT) for selective killing of cancer cells. Mol Ther.

[15] Amit D, Matouk IJ, Lavon I, Birman T, Galula J, Abu-Lail R, Schneider T, Siegal T, Hochberg A, Fellig Y (2012). Transcriptional targeting of glioblastoma by diphtheria toxin-A driven by both H19 and IGF2-P4 promoters. Int J Clin Exp Med.

[16] Lee EJ, Jameson JL (2002). Cell-specific Cre-mediated activation of the diphtheria toxin gene in pituitary tumor cells: potential for cytotoxic gene therapy. Hum Gene Ther.

[17] Massie B, Couture F, Lamoureux L, Mosser DD, Guilbault C, Jolicoeur P, Belanger F, Langelier Y (1998). Inducible overexpression of a toxic protein by an adenovirus vector with a tetracycline-regulatable expression cassette. J Virol.

[18] Wirth D, Gama-Norton L, Riemer P, Sandhu U, Schucht R, Hauser H (2007). Road to precision: recombinase-based targeting technologies for genome engineering. Curr Opin Biotechnol.

[19] Nafissi N, Slavcev R (2014). Bacteriophage recombination systems and biotechnical applications. Appl Microbiol Biotechnol.

[20] Gaj T, Sirk SJ, Barbas CF (2014). Expanding the scope of site-specific recombinases for genetic and metabolic engineering. Biotechnol Bioeng.

[21] Calos MP, Cathomen T, Hirsch M, Porteus M (2016). Genome Editing. Genome Editing: The Next Step in Gene Therapy.

[22] Meinke G, Bohm A, Hauber J, Pisabarro MT, Buchholz F (2016). Cre recombinase and other tyrosine recombinases. Chem Rev.

[23] Grindley N, Whitestone K, Rice P (2006). Mechanism of site-specific recombination. Annu Rev Biochem.

[24] Azaro MA, Landy A, Craig NL, Craigie R, Gellert M, Lambowitz A (2002). Integrase and the Lambda int family. Mobile DNAII.

[25] Weisberg RA, Gottesmann ME, Hendrix RW, Little JW (1999). Family values in the age of genomics: comparative analyses of temperate bacteriophage HK022. Annu Rev Genet.

[26] Harel-Levi G, Goltsman J, Tuby CN, Yagil E, Kolot M (2008). Human genomic site-specific recombination catalyzed by coliphage HK022 integrase. J Biotechnol.

[27] Malchin N, Goltsman J, Dabool L, Gorovits R, Bao Q, Droge P, Yagil E, Kolot M (2009). Optimization of coliphage HK022 Integrase activity in human cells. Gene.

[28] Lakso M, Pichel JG, Gorman JR, Sauer B, Okamoto Y, Lee E, Alt FW, Westphal H (1996). Efficient in vivo manipulation of mouse genomic sequences at the zygote stage. Proc Natl Acad Sci U S A.

[29] Elias A, Spector I, Sogolovsky-Bard I, Gritsenko N, Rask L, Mainbakh Y, Zilberstein Y, Yagil E, Kolot M (2016). Cancer-specific binary expression system activated in mice by bacteriophage HK022 Integrase. Sci Rep.

[30] Cong YS, Wen J, Bacchetti S (1999). The human telomerase catalytic subunit hTERT: organization of the gene and characterization of the promoter. Hum Mol Genet.

[31] Majumdar AS, Hughes DE, Lichtsteiner SP, Wang Z, Lebkowski JS, Vasserot AP (2001). The telomerase reverse transcriptase promoter drives efficacious tumor suicide gene therapy while preventing hepatotoxicity encountered with constitutive promoters. Gene Ther.

[32] Maxwell F, Maxwell IH, Glode LM (1987). Cloning, sequence determination, and expression in transfected cells of the coding sequence for the tox 176 attenuated diphtheria toxin A chain. Mol Cell Biol.

[33] Sauer B (1993). Manipulation of transgenes by site-specific recombination: use of Cre recombinase. Methods Enzymol.

[34] Loonstra A, Vooijs M, Beverloo HB, Allak B, van Drunen E, Kanaar R, Berns A, Jonkers J (2001). Growth inhibition and DNA damage induced by Cre recombinase in mammalian cells. Proc Natl Acad Sci U S A.

[35] Counter CM, Avilion AA, LeFeuvre CE, Stewart NG, Greider CW, Harley CB, Bacchetti S (1992). Telomere shortening associated with chromosome instability is arrested in immortal cells which express telomerase activity. EMBO J.

[36] Xi L, Schmidt JC, Zaug AJ, Ascarrunz DR, Cech TR (2015). A novel two-step genome editing strategy with CRISPR-Cas9 provides new insights into telomerase action and TERT gene expression. Genome Biol.

[37] Bertram JS, Janik P (1980). Establishment of a cloned line of Lewis Lung Carcinoma cells adapted to cell culture. Cancer Lett.

[38] Elmore S (2007). Apoptosis: a review of programmed cell death. Toxicol Pathol.

[39] Fridman JS, Lowe SW (2003). Control of apoptosis by p53. Oncogene.

[40] Weigel MT, Rath K, Alkatout I, Wenners AS, Schem C, Maass N, Jonat W, Mundhenke C (2014). Nilotinib in combination with carboplatin and paclitaxel is a candidate for ovarian cancer treatment. Oncology.

[41] Behrens A, Sabapathy K, Graef I, Cleary M, Crabtree GR, Wagner EF (2001). Jun N-terminal kinase 2 modulates thymocyte apoptosis and T cell activation through c-Jun and nuclear factor of activated T cell (NF-AT). Proc Natl Acad Sci U S A.

[42] Makena PS, Gorantla VK, Ghosh MC, Bezawada L, Balazs L, Luellen C, Parthasarathi K, Waters CM, Sinclair SE (1985). Lung injury caused by high tidal volume mechanical ventilation and hyperoxia is dependent on oxidant-mediated c-Jun NH2-terminal kinase activation. J Appl Physiol.

[43] Xia Y, Yeddula N, Leblanc M, Ke E, Zhang Y, Oldfield E, Shaw RJ, Verma IM (2012). Reduced cell proliferation by IKK2 depletion in a mouse lung-cancer model. Nat Cell Biol.

[44] Yu Y, Luk F, Yang JL, Walsh WR (2011). Ras/Raf/MEK/ERK pathway is associated with lung metastasis of osteosarcoma in an orthotopic mouse model. Anticancer Res.

[45] Hata AN, Engelman JA, Faber AC (2015). The BCL2 Family: Key Mediators of the Apoptotic Response to Targeted Anticancer Therapeutics. Cancer Discov.

[46] Adams JM, Cory S (2007). Bcl-2-regulated apoptosis: mechanism and therapeutic potential. Curr Opin Immunol.

[47] Luo M, Li L, Xiao C, Sun Y, Wang GL (2016). Heat stress impairs mice granulosa cell function by diminishing steroids production and inducing apoptosis. Mol Cell Biochem.

[48] Yang E, Korsmeyer SJ (1996). Molecular thanatopsis: a discourse on the BCL2 family and cell death. Blood.

[49] Oltvai ZN, Milliman CL, Korsmeyer SJ (1993). Bcl-2 heterodimerizes in vivo with a conserved homolog, Bax, that accelerates programmed cell death. Cell.

[50] Ries CH, Cannarile MA, Hoves S, Benz J, Wartha K, Runza V, Rey-Giraud F, Pradel LP, Feuerhake F, Klaman I, Jones T, Jucknischke U, Scheiblich S (2014). Targeting tumor-associated macrophages with anti-CSF-1R antibody reveals a strategy for cancer therapy. Cancer Cell.

[51] Katara GK, Kulshrestha A, Jaiswal MK, Pamarthy S, Gilman-Sachs A, Beaman KD (2016). Inhibition of vacuolar ATPase subunit in tumor cells delays tumor growth by decreasing the essential macrophage population in the tumor microenvironment. Oncogene.

[52] Todriya TV, Tzander A (2004). Telomerase activity in cells of 9-day-old spleen colonies formed by bone marrow of normal and thymectomized mice. Bull Exp Biol Med.

[53] Martin-Rivera L, Herrera E, Albar JP, Blasco MA (1998). Expression of mouse telomerase catalytic subunit in embryos and adult tissues. Proc Natl Acad Sci U S A.

[54] Hiyama E, Hiyama K (2003). Telomerase as tumor marker. Cancer Lett.

[55] Ramlee MK, Wang J, Toh WX, Li S (2016). Transcription regulation of the human telomerase reverse transcriptase (hTERT) gene. Genes.

[56] Baudino TA, Cleveland JL (2001). The Max network gone mad. Mol Cell Biol.

[57] Zhang F, Cheng D, Wang S, Zhu J (2016). Human specific regulation of the telomerase reverse transcriptase gene. Genes.

[58] Voziyanova E, Malchin N, Anderson RP, Yagil E, Kolot M, Voziyanov Y (2013). Efficient Flp-Int HK022 dual RMCE in mammalian cells. Nucleic Acids Res.

[59] Baba Y, Nakano M, Yamada Y, Saito I, Kanegae Y (2005). Practical range of effective dose for Cre recombinase-expressing recombinant adenovirus without cell toxicity in mammalian cells. Microbiol Immunol.

[60] Lyons SK, Meuwissen R, Krimpenfort P, Berns A (2003). The generation of a conditional reporter that enables bioluminescence imaging of Cre/loxP-dependent tumorigenesis in mice. Cancer Res.

[61] Mulay SV, Kim Y, Choi M, Lee DY, Choi J, Lee Y, Choi J, Lee Y, Jon S, Churchill DG (2018). Enhanced doubly-activated dual emission fluorescent probes for selective imaging of glutathione or cysteine in living systems. Anal Chem.

[62] Yuan N, Zhang X, Cao Y, Jiang X, Zhao S, Feng Y, Fan Y, Lu Z, Gao H (2017). Contrast-enhanced computerized tomography combined with a targeted nanoparticle contrast agent for screening for early-phase non-small cell lung cancer. Exp Ther Med.

[63] Bonnet ME, Erbacher P, Bolcato-Bellemin AL (2008). Systemic delivery of DNA or siRNA mediated by linear polyethylenimine (L-PEI) does not induce an inflammatory response. Pharm Res.

[64] Kawakami S, Ito Y, Charoensit P, Yamashita F, Hashida M (2006). Evaluation of proinflammatory cytokine production induced by linear and branched polyethylenimine/plasmid DNA complexes in mice. J Pharmacol Exp Ther.

[65] Chen X, Scapa JE, Liu DX, Godbey WT (2016). Cancer-specific promoters for expression-targeted gene therapy: ran, brms1 and mcm5. J Gene Med.

[66] Chen JS, Liu JC, Shen L, Rau KM, Kuo HP, Li YM Shi D, Lee YC, Chang KJ, Hung MC (2004). Cancer-specific activation of the survivin promoter and its potential use in gene therapy. Cancer Gene Ther.

[67] Dorgai L, Yagil E, Weisberg R (1995). Identifying determinants of recombination specificity: construction and characterization of mutant bacteriophage integrases. J Mol Biol.

[68] Sambrook J, Fritsch EF, Maniatis T (1989). Molecular cloning: a laboratory manual. 2 ed.

[69] Rask L, Fregil M, Hogdall E, Mitchelmore C, Eriksen J (2013). Development of a metastatic fluorescent Lewis Lung carcinoma mouse model: identification of mRNAs and microRNAs involved in tumor invasion. Gene.

[70] Kolot M, Meroz A, Yagil E (2003). Site-specific recombination in human cells catalyzed by the wild-type integrase protein of coliphage HK022. Biotechnol Bioeng.

