# Supplementary material for: Anti-cancer binary system activated by bacteriophage HK022 integrase
Source: Oncotarget. 2018 Jun 8;9(44):27487–501. doi: 10.18632/oncotarget.25512 (PMC6007955; doi:10.18632/oncotarget.25512)
Supplement: Supplementary file 1 [file oncotarget-09-27487-s001.pdf]

Anti-cancer binary system activated by bacteriophage HK022 integrase

SUPPLEMENTARY MATERIALS

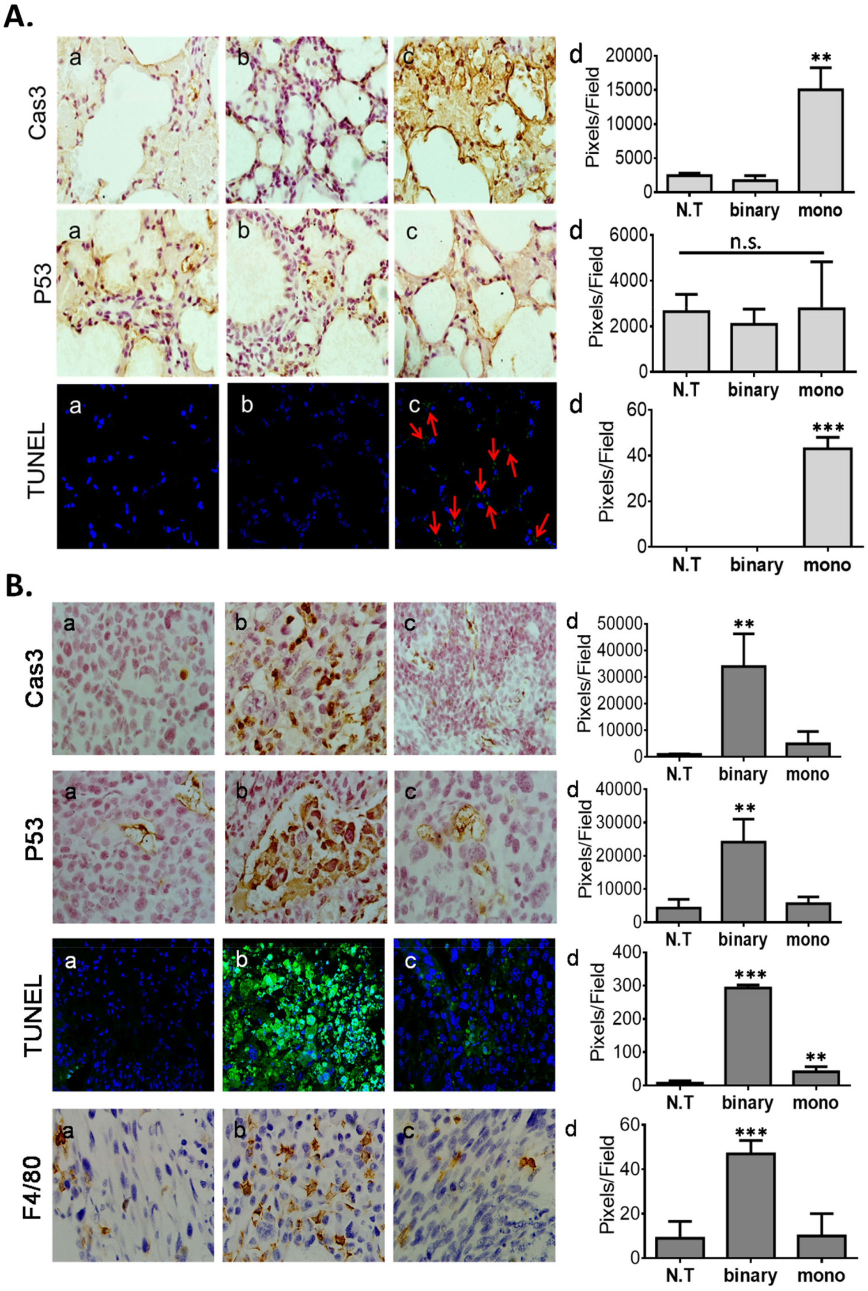

Supplementary Figure 1: IHC and qRT PCR analyses of the survival experiment mice lungs. (A) Healthy lungs. **(B)** LLC-Kat lungs. Cas-3, P53 (both brownish pigment) and TUNEL assay (green spots).(a) untreated mice (N.T.); (b) binary, (c) mono, (d) quantitative data. Each figure shows a typical lungs biopsy specimen from a cohort of at least six mice. The bars show the mean value of five experiments; the error bars indicate standard deviation.

(*Continued*)

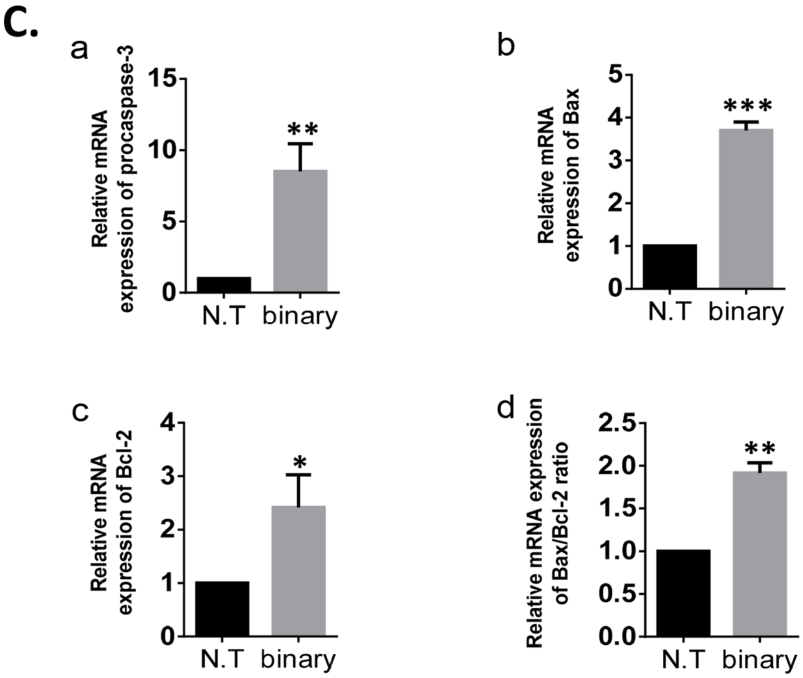

Supplementary Figure 1 (*Continued*): (C) qRT PCR analyses of RNA extracted from the cancer lungs of LLC-Kat mice from the survival experiment. The relative mRNA expression levels normalized to Katushka were detected for (a) procaspase-3, (b) Bax, (c) Bcl-2. (d) Bax to Bcl-2 ratio. n.s: not significant; ***p-val ≤ 0.001; **p-val ≤ 0.01; *p-val ≤ 0.05 vs. N.T.

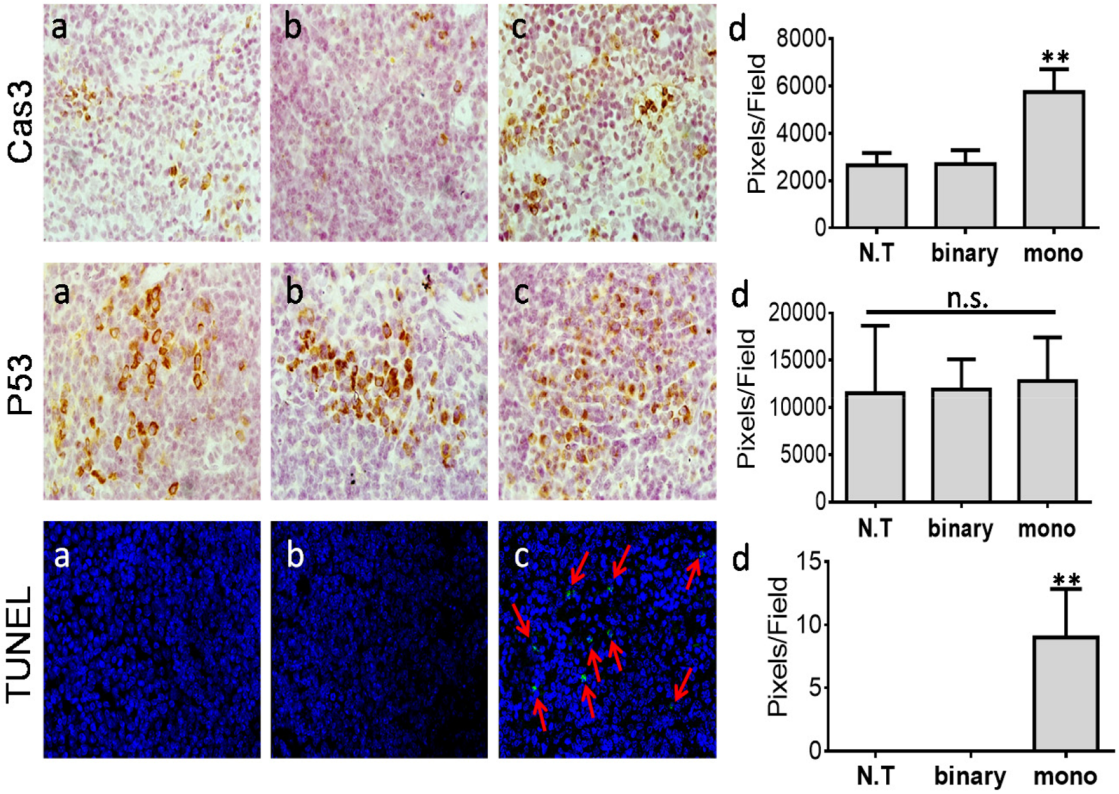

Supplementary Figure 2: IHC analyses of survival experiment healthy mice spleen. Cas-3, P53 (both brownish pigment) and TUNEL assay (green spots). (a) untreated mice (N.T.); (b) binary, (c) mono, (d) quantitative data. Each figure shows a typical lungs biopsy specimen from a cohort of at least six mice. The bars show the mean value of five experiments; the error bars indicate standard deviation. n.s: not significant; **p-val ≤ 0.01vs. N.T.

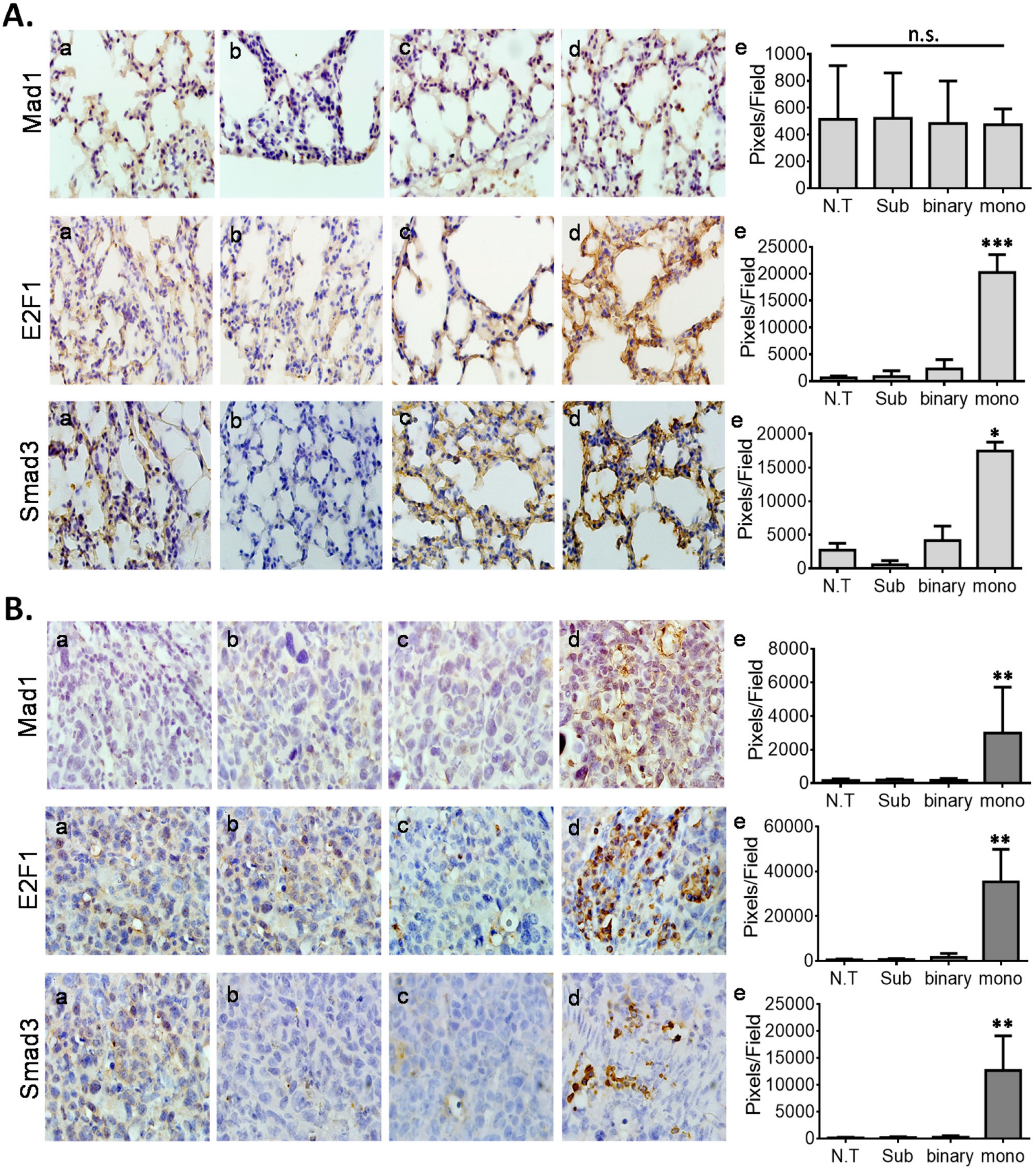

Supplementary Figure 3: IHC analyses of *TERT* promoter regulatory suppressing factors. (A) Healthy lungs. **(B)** LLC-Kat lungs. Mad1, E2F1 and Smad3(all brownish pigment). The mice were treated as the same conditions as in Figure 3. (a) untreated mice (N.T.), (b) Sub, (c) binary, (d) mono, (e) quantitative data. Each figure shows a typical lungs biopsy specimen from a cohort of at least five mice. The bars show the mean value of five experiments; the error bars indicate standard deviation. n.s: not significant; ***p-val ≤ 0.001; **p-val ≤ 0.01; *p-val ≤ 0.05 vs. N.T.
